# Feline haemotropic *Mycoplasma* spp. in Portugal: association with identifiable risk factors, clinical parameters and prognostic factors

**DOI:** 10.1186/s40575-026-00157-2

**Published:** 2026-09-23

**Authors:** Wenjun Li, Ana Teresa Severino Caldeira Reisinho, Luís Manuel Madeira de Carvalho

**Affiliations:** 1 Centre for Interdisciplinary Research in Animal Health, Faculty of Veterinary Medicine, Lisbon University, (CIISA-FMV-ULisboa), Avenida da Universidade Técnica, Lisboa, 1300-477 Portugal; 2 Associate Laboratory for Animal and Veterinary Sciences (AL4AnimalS), Lisbon, 1300-477 Portugal; 3 Veterinary Teaching Hospital, Faculty of Veterinary Medicine, Lisbon University (HEV-FMV-ULisboa) Avenida da Universidade Técnica, Lisboa, 1300-477 Portugal

**Keywords:** Cat, *Mycoplasma haemofelis*, Feline infectious anemia, Risk factors, Serology

## Abstract

**Objectives:**

This study aims to describe seroprevalence of feline haemotropic *Mycoplasma* spp. in a hospital population, and to analyze possible associations between recent infection and epidemiological and clinical characteristics of interest. Despite the inability to infer direct causality, statistical trends regarding identifiable factors that might impact the resolution of clinical cases were also explored.

**Methods:**

An analytical cross-sectional study was devised to analyze the relationship between recent feline haemotropic *Mycoplasma* spp. infection and epidemiological and clinical characteristics of interest. A total of 134 clinical cases were collected, consisting of cats treated at the Veterinary Teaching Hospital (HEV) of the Faculty of Veterinary Medicine, University of Lisbon, that underwent serological screening for haemotropic *Mycoplasma* spp. between January 1st, 2021, and June 30th, 2024. Potential relationships between a positive immunoglobulin M (IgM) test result and qualitative variables were studied using the Chi-Square test.

**Results:**

The prevalence of positive cases identified during the study period was 23.8% (32/134). A significant association between feline mycoplasmosis and FIV-positive cats and senior animals was observed, as well as a negative relationship between favorable clinical outcome and corticosteroid use. A tendency for positive animals to demonstrate anisocytosis and lymphadenopathy, but a negative relationship with low liver enzymes or total protein levels was also registered. No obvious correlation was observed between mycoplasmosis and the presence of anemia or ectoparasite infestations, confirming the complexity of its clinical and epidemiological profile.

**Clinical significance:**

The results of this study confirm the presence and circulation of this disease’s agents in the feline population treated at a veterinary hospital in Lisbon and reinforce the need to adjust the assessment and clinical approach of a positive diagnosis according to the patient’s overall condition, plus the importance of monitoring cases with potential for disease reactivation.

## Lay summary

This study was focused on feline haemotropic *Mycoplasma*, a group of bacteria that can infect cats through direct means (social contact with other cats) or vectors such as fleas and ticks, capable of causing red blood cell destruction. Cats presenting to consultation at a Lisbon Veterinary hospital with suspicion of infection were tested for a class of antibodies that indicate a relatively recent contact with these bacteria. These test results were then cross-referenced with their clinical data and lifestyle factors. Out of 132 cats 32 (23.8%) tested positive to recent infection, which were more commonly associated with older age and FIV (feline immunodeficiency virus) positive patients. There was no clear relationship between *Mycoplasma* infection and anaemia or flea presence, as positive cats can be clearly anemic or have seemingly normal blood test results. Seropositive cats were more likely to present a bigger range of red blood cell sizes and some tendency to display reactive lymph nodes. Anaemic cats that needed treatment with corticosteroids were less likely to have a favourable clinical outcome. This study shows that haemotropic *Mycoplasma* infections are prevalent among a sample of urban cats and that a positive test result should always be analyzed with the cat’s overall clinical presentation as some cases can be asymptomatic carriers while others develop anemia and require appropriate medical care. In some situations, cats may need ongoing monitoring due to the possibility that infection can persist even after an appropriate course of antibiotic treatment.

## Introduction

Haemotropic *Mycoplasma* spp. (also known as haemoplasmas) are bacteria that exhibit tropism for vertebrate host erythrocytes, with the potential to cause hemolytic anemia in affected hosts [1–3]. These bacteria belong to the *Mycoplasma* genus, with three species recognized worldwide as agents of feline infectious anemia: *Mycoplasma haemofelis*, “*Candidatus* Mycoplasma haemominutum”, and “*Candidatus* Mycoplasma turicensis” [4, 5].

Despite some geographical variations, the agents of feline mycoplasmosis have a worldwide distribution (Table 1), with records of their detection in domestic, stray, and wild cats [6–10]. Most studies report a higher prevalence of “*Candidatus* Mycoplasma haemominutum” (“*Ca*. M. haemominutum”), followed by *Mycoplasma haemofelis* (*M. haemofelis*), and in third place “*Candidatus* Mycoplasma turicensis” (“*Ca*. M. turicensis”) [11–14].

**Table 1:** International prevalence studies of feline mycoplasmosis

Country	Year	n	*M. haemofelis*	“*Ca*. M. haemominutum”	“*Ca*. M. turicensis”	*Mycoplasma* spp.
Iran [15]	2024	361	2.2%	10.5%	0.6%	15.5%
Spain [16]	2023	332	10.5%	18.4%	3.3%	22.9%
China [17]	2021	668	0.9%	3.4%	1.2%	4.9%
Thailand [18]	2020	231	3.9%	22.9%	0.86%	27.7%
Italy [13]	2020	958	1.5%	9.9%	0.2%	11.6%
Brazil [19]	2018	200	12%	12.5%	3%	35.5%
Cyprus [20]	2017	174	7.5%	20.7%	6.9%	26.4%
Chile [21]	2016	384	4.4%	7.8%	2%	15.1%
Turkey [22]	2016	384	9.9%	17.7%	0.8%	19.3%
Portugal [11]	2015	236	14.4%	17.8%	5.9%	27.1%
Albania [23]	2014	146	10.3%	21.9%	5.5%	30.8%
South Africa [24]	2012	102	3.9%	21.6%	0%	25.5%
Japan [25]	2010	1770	2.4%	15.8%	2.7%	26.4%
South Korea [26]	2007	331	4.2%	10.3%	0%	19.9%

Coinfection by more than one agent in the same host is frequent, due to these agents having similar risk factors and transmission routes, with *M. haemofelis* paired with “*Ca*. M. haemominutum” being the most common combination [14, 27–30].

These agents are also documented in many species of wild felids, whether free-ranging or kept in captivity (lions, tigers, cheetahs, Iberian lynxes, leopards, jaguars, cougars, etc.), with “*Ca*. M. haemominutum” being the most prevalent species and records of mixed infections, similar to domestic cats [31–35].

A higher prevalence of infection is usually identified in male cats with access to the outdoors, with their ambulatory and territorial behavior being pointed out as a possible cause for the increased likelihood of aggressive interactions with other cats and greater exposure to arthropod vectors [16, 36–39]. It has also been noted on occasions that animals identified as positive for feline immunodeficiency virus (FIV) or feline leukemia virus (FeLV) have a higher risk of co-infection with feline *Mycoplasma* spp., with special emphasis on FIV, which is also strongly associated with transmission through aggressive interactions [7, 40–44].

The most commonly reported clinical signs are usually nonspecific such as lethargy and anorexia, but also include an assortment of signs that reflect the severity of the underlying anemia, such as pale mucous membranes, tachycardia, splenomegaly, jaundice, heart murmurs on auscultation, among others [8, 45–47]. Occasionally, no obvious relationship between feline mycoplasmosis and anemia is found due to the existence of completely asymptomatic chronic carriers [48–50]. For patients who do develop acute anemia, it is usually regenerative (macrocytic and hypochromic), and may be accompanied by anisocytosis, reticulocytosis, and polychromasia [21, 48, 51, 52]. Platelet counts may be normal or slightly subnormal [53, 54]. There may also be increases in liver enzymes due to hepatic hypoxia, hyperbilirubinemia due to hemolysis, some increase in total protein, or albumin/globulin ratio shifts due to polyclonal gammopathy [14, 55–57].

Cytological diagnosis of feline haemoplasmas is an accessible but low sensitivity method, as it usually requires exuberant bacteremia to be visualized by experienced operators and does not allow differentiation of the three species due to their morphological similarities [5, 42, 53, 58]. In comparison, polymerase chain reaction (PCR) has demonstrated high sensitivity and specificity for detecting fragments of the 16 S rRNA gene of *Mycoplasma* spp. in different types of samples such as saliva, blood, urine, feces, among others [5, 59–61]. However, the amount of haemoplasma determined does not directly correlate with the animal’s clinical status, and there are cases that remain persistently seropositive despite being negative by PCR, so there’s a presumed persistence of haemoplasmas at levels below the PCR detection limit in these situations [62–66]. Alternatively, serological techniques are a practical and convenient diagnostic method, thanks to the existence of specific antibodies whose titers reflect the progression of the infection, with IgM titers allowing the distinction between acute and chronic infections [61, 67–69].

The aim of this study was to evaluate the prevalence of feline *Mycoplasma* spp. diagnosed in patients at the HEV (Veterinary Teaching Hospital), as well as to compare negative and recently infected cats in terms of risk factors, clinical signs and laboratory parameters. Factors likely to influence the prognosis of positive cases were also analyzed.

## Materials and methods

### Study design and inclusion criteria

An analytical cross-sectional study was devised with the target population set as HEV feline patients who underwent feline mycoplasmosis screening, between January 1st, 2021, and June 30th, 2024.

The criterion used to classify a case as an acute feline mycoplasmosis infection was the presence of a positive Immunoglobulin M (IgM) result. Cases diagnosed by PCR were not included due to a shortage of cases screened by this method during the study period. Patients who only had a positive Immunoglobulin G (IgG) result, but no IgM information were also excluded [56, 68, 70].

Not all cases had a complete blood count and/or extensive biochemical analyses available at the time of haemoplasma infection diagnosis, and some clinical records did not allow for the complete collection of information such as vaccination status, FIV/FeLV status, frequency of internal and external parasite prophylaxis, among others. The data obtained were analyzed with the awareness that there is missing information.

#### Variables of interest

Based on previous studies that dwelled onto the epidemiological aspects of feline haemotropic *Mycoplasma* infection [5, 39, 44, 71], namely the possibility of horizontal and/or vector borne transmission, extra care was taken when collecting anamnesis information regarding each cat’s lifestyle to evaluate their epidemiological risk. Profile information was collected regarding their origins (adopted from breeder or street rescue), whether they lived in multicat households, outdoor access (to assess exposure to vectors or interspecies aggression), fastidiousness of vaccination and external parasiticide application, FIV/FeLV status, as well as any other useful information that could be obtained from their medical records. When applicable, any pre-existing conditions such as concomitant co-morbidities or co-infections were logged to assess their statistical usefulness later.

Another point of interest was to inventory clinical and analytical findings during the diagnostic work-up so any available information regarding clinical signs (such as depression, pale mucous membranes, tachycardia/tachypnea, jaundice, fever, etc.) and routine complete blood count and biochemistry panels performed around the time of *Mycoplasma* spp. screening were also recorded for analysis. Clinical and analytical data that were chronologically too distant from the moment of *Mycoplasma* spp. screening were considered unfit for statistical analysis.

Finally, study patients’ clinical outcomes and therapy details were also documented, such as cases that actually started treatment (with antibiotics, corticosteroids, blood transfusions, etc.) while other cats were euthanized. These treatment details, along with any logged co-morbidities and co-infections, were thought to be an interesting aspect to explore in an attempt to identify any visible trend with the clinical outcome of the case.

### Medical record search

Patient information was collected by retrieving their clinical records and organized into a database containing their profile (sex, age, environment, lifestyle, vaccination status, internal and external parasite prophylaxis, medical and surgical history). After verifying the complete clinical history of each patient, blood count values and biochemical analyses were collected for the period in which clinical suspicion of feline mycoplasmosis arose. Part of the patients had complete blood count and biochemistry analyses requested simultaneously with etiological diagnosis, but some cases underwent haemoplasma screening with a slight delay, in which cases a maximum difference of 72 h was accepted. In addition to blood count information, other data were collected such as blood urea nitrogen (BUN), creatinine, aspartate aminotransferase (AST), alanine aminotransferase (ALT), alkaline phosphatase (ALP), total protein, albumin, globulins, albumin/globulin ratio, and total bilirubin.

Relevant clinical information was also extracted along the timeline of feline mycoplasmosis screening, such as clinical signs observed during consultation (fever, pale mucous membranes, tachypnea/tachycardia, etc.), administered treatment, and outcome of each case.

Follow-up information was scoured until the most recent entries for each patient, with some patients returning for reevaluation while others had no further entries after medical discharge.

### Data extracted

The data collected from clinical records were organized and sorted into a database, summarized in Table 2.

**Table 2:** Organization and classification of collected variables

Variable	Classification	Reference range
IgM indirect immunofluorescence	Positive or negative result	
IgG indirect immunofluorescence	Positive or negative result	
Sex	Male or female	
Reproductive status	Intact or neutered	
Season of the year (Time of the year when feline mycoplasmosis screening was performed)	Winter	December 21st to March 20th
	Spring	March 21st to June 20th
	Summer	June 21st to September 22nd
	Autumn	September 23rd to December 20th
Breed	Pure or mixed breed	
Age range	Junior	< 1 year old
	Adult	1–10 years old
	Senior	> 10 years old
Age	Expressed as years	
Other Vector Borne Pathogens (VBPs)	Presence or absence of other diagnosed VBPs (*Ehrlichia canis*,* Rickettsia conorii*,* Leishmania infantum*, etc.)	
Ectoparasites	If ectoparasites such as fleas and/or ticks were found during the physical examination	
Internal parasiticide	Regular or inconsistent use of internal parasiticides	
External parasiticide	Regular or inconsistent use of external parasiticides	
FIV status	Positive or negative for feline immunodeficiency retrovirus	
FeLV status	Positive or negative for feline leukemia retrovirus	
Vaccination status	Up-to-date vaccinations or if vaccinations are overdue	
Living environment	With or without outdoor access	
Rescued stray	Sorted based on whether they were previously stray cats rescued from the street or not	
Number of cohabitants	Whether it’s the only pet at home or a household with multiple pets	
Body weight	Body weight in kg	
Hematocrit	Presence or absence of anemia	24–45%
Reticulocytes	Classification of anemia as regenerative or non-regenerative	3000–50,000/µL; considered regenerative if > 42,000/µL
Mean Corpuscular Volume (MCV)	Classification of anemia as macrocytic, normocytic, or microcytic	39–52 fL
Mean Corpuscular Hemoglobin (MCH)	Classification of anemia as normochromic or hypochromic	12.5–17.5 pg
Red Cell Distribution Width (RDW)	Presence or absence of anisocytosis	17–23%
Leukocytes	Leukocytosis, leucopenia or normal count	5.5–17.02 × 10³/mm³
Lymphocytes	Lymphocytosis, lymphopenia, or normal count	1.5–7 × 10³/mm³
Monocytes	Monocytosis or normal count	0–1400/mm³
Neutrophils	Neutrophilia, neutropenia or normal count	2.5–12.8 × 10³/mm³
Eosinophils	Eosinophilia or normal count	0–1.5 × 10³/mm³
Platelets	Thrombocytopenia or normal count	150–500 × 10³/mm³
BUN	High values or values within the normal range	30–65 mg/dL
Creatinine	High values or values within the normal range	1.1–2.3 mg/dL
AST	High values or values within the normal range	8–35 U/L
ALT	Low, high or values within the normal range	33–119 U/L
ALP	High values or values within the normal range	7–68 U/L
Total protein	Hyperproteinemia, hypoproteinemia or normal values	6.4–8.5 g/dL
Albumin	Hypoalbuminemia or normal values	3.1–4.3 g/dL
Globulins	Abnormal values or within the reference range	2.8–5.1 g/dL
Albumin/globulin ratio	Abnormal values or within the reference range	0.45–1.3
Total bilirubin	Abnormal values or within the reference range	0–0.4 mg/dL
Coagulation tests	Abnormal values or within the reference range	Prothrombin time: 7–10 s Thrombin time: 11.6–15.7 s Partial thromboplastin time: 8.6–12.9 s
Coombs test	Positive or negative result	
Clinical signs	Parameters recorded during physical examination such as prostration, hyporexia, fever, hypothermia, pale mucous membranes, signs of jaundice, abnormalities detected by cardiopulmonary auscultation, evident tachypnea or tachycardia, etc.	
Non-vectorial diseases	Variable that includes other concomitant diseases/medical-surgical history that were not the reason for feline *Mycoplasma* screening (dermatological, renal, hepatobiliary, oncological disease, etc.)	
Treatment	If targeted antibiotic therapy was needed for feline mycoplasmosis	
Doxycycline	If there was a need to institute doxycycline therapy	
Marbofloxacin	If there was a need to institute therapy with marbofloxacin	
Corticosteroids	If there was need to combine corticosteroid therapy with antibiotic therapy	
Blood transfusion	If transfusion with packed red blood cells or fresh frozen plasma was necessary	
Outcome	Whether the clinical case was solved or the patient died/was euthanized during the course of feline mycoplasmosis	

### Data analysis

Statistical analysis of the collected data was performed using Microsoft Office Excel (version 2409), Jamovi (version 2.6.44.0), and IBM SPSS Statistics^®^ (version 29). The established statistical significance value was *p* ≤ 0.05 for all tests performed.

Categorical variables were sorted into appropriate groups (with or without outdoor access, IgM positive or negative, with or without jaundice, etc.) while quantitative variables such as complete blood count and biochemistry values were classified as below, above or within physiological parameters according to the outsourced laboratory’s set reference ranges.

To calculate the prevalence, the number of IgM-positive cats was divided by the total number of the sample, reported as a percentage with a 95% confidence interval. Quantitative variables such as weight and age of the cats were reported as mean and standard deviation.

The existence of any relationship between a positive IgM result and qualitative variables was studied using the Chi-Square test, or Fisher’s exact test in cases where sample size does not meet the requirements of the Chi-Square test. Odds Ratio (OR) with a 95% confidence interval (95% CI) was also determined.

Categorical variables were divided into three main sections for analysis: relationship between IgM status and possible risk factors; relationship between IgM status and clinical signs or analytical changes (complete blood count and biochemistry parameters); relationship between clinical outcome and possible prognostic factors (administered medication, concomitant diseases, etc.).

A first approach univariate analysis was performed on each group of variables under study, and variables that demonstrated a *p*-value < 0.2 were subsequently selected to assess their value in the construction of multivariate analysis models. This type of analysis assesses the simultaneous effect of a set of independent variables on a dependent variable (often designated as the “outcome”), allowing the assessment of causal correlations and estimating their degree of effect on the response variable [72, 73].

In situations where the outcome variable is expressed quantitatively, a linear regression model would have been used, but in the present study, given that the dependent variable is binary in nature (IgM positive or negative; positive or negative clinical outcome), logistic regression models were constructed [72, 74].

## Results

By searching the laboratory database and applying the aforementioned inclusion and exclusion criteria, it was possible to identify 134 viable cases that underwent serology testing for *Haemobartonella* or *Mycoplasma* spp. Due to the reduced number of cases that were simultaneously screened for other vector-borne pathogens (VBPs) (5 positive for *Ehrlichia*, 2 positive for *Rickettsia*, and none positive for *Leishmania*), it was not possible to analyze their relationship with feline mycoplasmosis. Similarly, other variables that were not analyzed due to the limited number of recorded cases included: cases that received blood transfusion during their hospitalization, patients with concurrent cardiac disease, patients with concurrent kidney disease, and patients that had a Coombs test performed.

### Characterization of the study animals

The average age of the selected cats was 7.4 ± 5 years. The average weight of the cats was 4.3 ± 1.3 kg. Of the 134 cats, 74 were identified as male and 60 as female. Most cats had been previously neutered (114/134, 85%). A large proportion of the cats were described as European Shorthair (59/134, 44%) or mixed breed (55/134, 41%). Other less frequent breeds (20/134, 15%) were also identified, such as Persian, Norwegian Forest Cats, British Shorthair, Scottish Fold, etc.

A large proportion of the cats were described as indoor cats (103/134, 76.9%) while the remainder were classified as cats with access to the outdoors. A portion of the cats lived with other pets at home (84/134, 62.7%). Some of the animals were described as previous strays rescued from the street (37/134, 27.6%).

Regarding vaccination, most cats had up to date vaccination status (80/122, 65.6%), but inconsistent internal (81/103, 78.6%) and external (78/104, 75%) parasite prophylaxis habits. Ectoparasites were found in 31 (23%) of the cases studied, with fleas being the most frequent finding. Upon physical examination, none of the cats presented wounds or other lesions that would suggest recent aggression.

Regarding clinical outcome of the study cases, some of the cats (30/134, 22.4%) passed away or were euthanized upon owner request while majority (104/134, 77.6%) of them achieved clinical recovery.

### Observed prevalence

Of the 134 cats tested for haemoplasma at the HEV, 32 had positive IgM results, while 102 had negative IgM results, therefore the observed prevalence was 23.8% (95% CI: 0.169–0.320).

### Analysis of the relationship between haemoplasma infection and risk factors

The results of univariate and multivariate analysis of risk factors were summarized in Tables 3 and 4, respectively. Univariate analysis identified a statistically significant association between FIV-positive animals and positive IgM status (OR of 13.3, *p* < 0.001). Multivariate analysis confirmed this association of haemoplasma infection with FIV-positive animals (adjusted OR of 12.768, *p* = 0.05), as well as a negative association between haemoplasma infection and the senior age group (adjusted OR of 0.333, *p* = 0.043). No statistically significant differences were identified for other analyzed variables.

**Table 3:** Univariate analysis of the relationship between haemoplasma infection and risk factors

Risk factor	*n*	IgM negative (% row)	IgM positive (% row)	OR	95% CI	*p*
Sex	134					
Male	74	57 (77)	17 (23)	1.118	0.504–2.479	0.784
Female	60	45 (75)	15 (25)			
Reproductive status	134					
Neutered	114	88 (77.2)	26 (22.8)	1.45	0.507–4.15	0.486
Intact	20	14 (70)	6 (30)			
Season	134					
Winter	33	23 (69.7)	10 (30.3)			0.754
Spring	42	33 (78.6)	9 (21.4)			
Summer	35	28 (80)	7 (20)			
Autumn	24	18 (75)	6 (25)			
Breed	134					
Pure breed	81	63 (77.8)	18 (22.2)	1.256	0.562–2.809	0.578
Mixed breed	53	39 (73.6)	14 (26.4)			
Age range (F)	134					
Junior	7	6 (85.7)	1 (14.3)			0.14*
Adult	80	56 (70)	24 (30)			
Senior	47	40 (85.1)	7 (14.9)			
Ectoparasites	134					
No	103	76 (73.8)	27 (26.2)	0.541	0.189–1.55	0.248
Yes	31	26 (83.9)	5 (16.1)			
Internal parasiticide (F)	103					
Regular	22	18 (81.8)	4 (18.2)	1.379	0.416–4.575	0.775
Inconsistent	81	62 (76.5)	19 (23.5)			
External parasiticide	104					
Regular	26	19 (73.1)	7 (26.9)	0.756	0.273–2.097	0.591
Inconsistent	78	61 (78.2)	17 (21.8)			
FIV (F)	124					
Negative	115	91 (79.1)	24 (20.9)	13.3	2.59–68	< 0.001*
Positive	9	2 (22.2)	7 (77.8)			
FeLV (F)	124					
Negative	118	88 (74.6)	30 (25.4)	0.587	0.066–5.22	1
Positive	6	5 (83.3)	1 (16.7)			
Vaccination	122					
Regular	80	63 (78.8)	17 (21.3)	1.158	0.479–2.819	0.746
Inconsistent	42	32 (76.2)	10 (23.8)			
Living environment	134					
Indoor	103	80 (77.7)	23 (22.3)	1.423	0.576–3.512	0.443
Outdoor	31	22 (71)	9 (29)			
Rescued stray	134					
No	97	77 (79.4)	20 (20.6)	1.85	0.793–4.31	0.152*
Yes	37	25 (67.6)	12 (32.4)			
Cohabitants	134					
Sole pet	50	36 (72)	14 (28)	0.701	0.313–1.573	0.388
More pets	84	66 (78.6)	18 (21.4)			

* Symbolizes variables selected for multivariate analysis; (F) symbolizes variables that were analyzed with Fisher’s exact test; bold values symbolize *p* ≤ 0.05

**Table 4:** Multivariate logistic regression analysis of the relationship between haemoplasma infection and risk factors

Risk factor	OR	95% CI	*p*
Age range			
Junior - Adult	0.256	0.021–3.12	0.285
Adult - Senior	0.333	0.115–0.964	**0.043**
FIV	12.768	2.167–75.225	**0.005**
Rescued stray	1.519	0.567–4.067	0.406

Reference categories: FIV = negative, rescued stray = negative, age range = adult; bold values represent *p* ≤ 0.05

### Analysis of the relationship between haemoplasma infection and clinical/laboratory parameters

The results of univariate analysis of hematological, biochemical, and clinical variables are summarized in Tables 5, 6, and 7, respectively. No statistically significant differences were identified even after multivariate analysis of variables that showed *p* < 0.2 in the univariate analysis (Table 8). However, a prominent association was observed between IgM-positive animals and certain variables, such as red blood cell distribution width (adjusted OR of 2.588, *p* = 0.119) and lymphadenopathy (adjusted OR of 7.31, *p* = 0.126). Conversely, a negative relationship was observed between feline mycoplasmosis and cases with low ALT (adjusted OR of 0.209, *p* = 0.157) and cases of hypoproteinemia (adjusted OR of 0.374, *p* = 0.243).

**Table 5:** Univariate analysis of the relationship between haemoplasma infection and hematological parameters

Hematological parameter	*n*	IgM negative (% row)	IgM positive (% row)	OR	95% CI	*p*
Hematocrit	130					
Normal	74	57 (77)	17 (23)	1.01	0.445–2.31	0.974
Anemia	56	43 (76.8)	13 (23.2)			
Reticulocytes (F)	74					
Regenerative	10	8 (80)	2 (20)	1.818	0.354–9.346	0.713
Non-regenerative	64	44 (68.8)	20 (31.3)			
MCV (F)	130					
Normocytic	98	78 (79.6)	20 (20.4)			0.386
Macrocytic	14	10 (71.4)	4 (28.6)			
Microcytic	18	12 (66.7)	6 (33.3)			
MCH (F)	130					
Normochromic	119	92 (77.3)	27 (22.7)	1.278	0.317–5.153	0.715
Hypochromic	11	8 (72.7)	3 (27.3)			
RDW	116					
Normal	57	48 (84.2)	9 (15.8)	2.159	0.871–5.352	0.093*
Anisocytosis	59	42 (71.2)	17 (28.8)			
Leukocytes	130					
Normal	82	66 (80.5)	16 (19.5)			0.227
Leukocytosis	23	18 (78.3)	5 (21.7)			
Leukopenia	25	16 (64)	9 (36)			
Lymphocytes (F)	130					
Normal	61	50 (82)	11 (18)			0.368
Lymphocytosis	5	4 (80)	1 (20)			
Lymphopenia	64	46 (71.9)	18 (28.1)			
Monocytes (F)	130					
Normal	123	96 (78)	27 (22)	2.667	0.562–12.648	0.2*
Monocytosis	7	4 (57.1)	3 (42.9)			
Neutrophils (F)	130					
Normal	90	70 (77.8)	20 (22.2)			0.551
Neutrophilia	25	20 (80)	5 (20)			
Neutropenia	15	10 (66.7)	5 (33.3)			
Eosinophils (F)	130					
Normal	128	99 (77.3)	29 (22.7)	3.414	0.207–56.281	0.41
Eosinophilia	2	1 (50)	1 (50)			
Platelets	130					
Normal	99	76 (76.8)	23 (23.2)	0.964	0.368–2.52	0.94
Thrombocytopenia	31	24 (77.4)	7 (22.6)			

* Symbolizes variables selected for multivariate analysis; (F) represents variables that were analyzed with Fisher’s exact test; bold values represent *p* ≤ 0.05

**Table 6:** Univariate analysis of the relationship between haemoplasma infection and biochemical parameters

Biochemical parameter	*n*	IgM negative (% row)	IgM positive (% row)	OR	95% CI	*p*
BUN	121					
Normal	83	61 (73.5)	22 (26.5)	0.52	0.191–1.412	0.195*
High	38	32 (84.2)	6 (15.8)			
Creatinine (F)	124					
Normal	106	81 (76.4)	25 (23.6)	0.926	0.279–3.068	1
High	18	14 (77.8)	4 (22.2)			
AST (F)	22					
Normal	12	11 (91.7)	1 (8.3)	4.714	0.405–54.826	0.293
High	10	7 (70)	3 (30)			
ALT (F)	121					
Normal	85	62 (72.9)	23 (27.1)			0.084*
Low	20	19 (95)	1 (5)			
High	16	13 (81.3)	3 (18.8)			
ALP	91					
Normal	65	50 (76.9)	15 (23.1)	1.481	0.538–4.080	0.446
High	26	18 (69.2)	8 (30.8)			
Total protein	99					
Normal	67	48 (71.6)	19 (28.4)			0.169*
Hypoproteinemia	26	23 (88.5)	3 (11.5)			
Hyperproteinemia	6	4 (66.7)	2 (33.3)			
Albumin	117					
Normal	78	62 (79.5)	16 (20.5)	1.722	0.718–4.129	0.22
Hypoalbuminemia	39	27 (69.2)	12 (30.8)			
Globulins	95					
Normal	74	55 (74.3)	19 (25.7)	0.905	0.292–2.805	0.862
Abnormal	21	16 (76.2)	5 (23.8)			
Albumin/globulin ratio (F)	95					
Normal	86	62 (72.1)	24 (27.9)	0.721	0.632–0.822	0.106*
Abnormal	9	9 (100)	0 (0)			
Bilirubin (F)	42					
Normal	20	15 (75)	5 (25)	0.882	0.213–3.653	1
Abnormal	22	17 (77.3)	5 (22.7)			
Coagulation tests (F)	10					
Normal	6	5 (83.3)	1 (16.7)	0.407	0.013–12.6	1
Abnormal	4	4 (100)	0 (0)			

* Symbolizes variables selected for multivariate analysis; (F) represents variables that were analyzed with Fisher’s exact test; bold values represent *p* ≤ 0.05

**Table 7:** Univariate analysis of the relationship between haemoplasma infection and clinical signs

Clinical signs	*n*	IgM negative (% row)	IgM positive (% row)	OR	95% CI	*p*
Prostration	134					
No	61	48 (77.4)	14 (22.6)	1.14	0.514–2.54	0.743
Yes	72	54 (75)	18 (25)			
Mucous membranes	134					
Normal	89	65 (73)	24 (27)	0.586	0.239–1.43	0.239
Pale	45	37 (82.2)	8 (17.8)			
Jaundice	134					
No	111	84 (75.7)	27 (24.3)	0.864	0.293–2.55	0.791
Yes	23	18 (78.3)	5 (21.7)			
Weight loss (F)	134					
No	116	89 (76.7)	27 (23.3)	1.27	0.415–3.88	0.767
Yes	18	13 (72.2)	5 (27.8)			
Fever	134					
No	98	75 (76.5)	23 (23.5)	1.09	0.448–2.64	0.854
Yes	36	27 (75)	9 (25)			
Hypothermia (F)	134					
No	125	95 (76)	30 (24)	0.905	0.178–4.59	1
Yes	9	7 (77.8)	2 (22.2)			
Cardiopulmonary auscultation	134					
Normal	90	69 (76.7)	21 (23.3)	1.1	0.473–2.53	0.832
Murmurs	44	33 (75)	11 (25)			
Hyporexia	134					
No	52	41 (78.8)	11 (21.2)	1.28	0.560–2.94	0.555
Yes	82	61 (74.4)	21 (25.6)			
Tachycardia (F)	134					
No	121	94 (77.7)	27 (22.3)	2.18	0.658–7.2	0.301
Yes	13	8 (61.5)	5 (38.5)			
Tachypnea (F)	134					
No	121	90 (74.4)	31 (25.6)	0.242	0.03–1.94	0.189*
Yes	13	12 (92.3)	1 (7.7)			
Lymphadenopathy (F)	134					
No	127	99 (78)	28 (22)	4.71	0.996–22.3	0.056*
Yes	7	3 (42.9)	4 (57.1)			

* Symbolizes variables selected for multivariate analysis; (F) represents variables that were analyzed with Fisher’s exact test; bold values represent *p* ≤ 0.05

**Table 8:** Multivariate logistic regression analysis of the relationship between feline mycoplasmosis and clinical/laboratory parameters

Variable	OR	95% CI	*p*
RDW	2.588	0.783–8.557	0.119
Monocytes	0.916	0.111–7.562	0.935
BUN	0.85	0.244–2.967	0.799
ALT			
Low - Normal	0.209	0.024–1.826	0.157
High - Normal	0.809	0.168–3.892	0.792
Total protein			
Hypoproteinemia - Normal	0.374	0.072–1.948	0.243
Hyperproteinemia - Normal	0.889	0.074–10.758	0.926
Tachypnea	0.4	0.041–3.912	0.431
Lymphadenopathy	7.31	0.572–93.47	0.126

Reference categories: Normal/Negative

### Analysis of the relationship between prognostic factors and outcome of haemoplasma infection cases

The results of univariate and multivariate analysis of prognostic factors were summarized in Tables 9 and 10. Multivariate analysis revealed a negative correlation between a favorable prognosis and the use of corticosteroids (adjusted OR of 0.068, *p* = 0.05). Although not statistically significant, the univariate analysis showed a positive trend between animals with favorable clinical resolution and the use of doxycycline (OR of 3.71, *p* = 0.355), while variables such as the presence of hepatic disease (OR of 0.12, *p* = 0.105) and oncologic disease (OR of 0.154, *p* = 0.292) showed an inverse relationship with a positive clinical outcome.

**Table 9:** Univariate analysis of the relationship between prognostic factors and clinical outcome

Prognostic factors	*n*	Euthanasia/death (% row)	Recovered (% row)	OR	95% CI	*p*
Treatment (F)	32					
No	4	1 (25)	3 (75)	2	0.164–24.3	0.512
Yes	28	4 (14.3)	24 (85.7)			
Doxycycline (F)	32					
No	18	4 (22.2)	14 (77.8)	3.71	0.366–37.7	0.355
Yes	14	1 (7.1)	13 (92.9)			
Marbofloxacin (F)	32					
No	16	1 (6.3)	15 (93.8)	0.2	0.02–2.03	0.333
Yes	16	4 (25)	12 (75)			
Corticosteroids (F)	32					
No	27	3 (11.1)	24 (88.9)	0.188	0.022–1.62	0.163*
Yes	5	2 (40)	3 (60)			
Dermatologic (F)	32					
No	29	4 (13.8)	25 (86.2)	0.32	0.023–4.41	0.41
Yes	3	1 (33.3)	2 (66.7)			
Renal (F)	32					
No	27	5 (18.5)	22 (81.5)	2.69	0.128–56.3	0.564
Yes	5	0 (0)	5 (100)			
Hepatic (F)	32					
No	28	3 (10.7)	25 (89.3)	0.12	0.012–1.19	0.105*
Yes	4	2 (50)	2 (50)			
Oncologic (F)	32					
No	30	4 (13.3)	26 (86.7)	0.154	0.008–2.98	0.292
Yes	2	1 (50)	1 (50)			

* Symbolizes variables selected for multivariate analysis; (F) represents variables that were analyzed with Fisher’s exact test; bold values represent *p* ≤ 0.05

**Table 10:** Multivariate logistic regression analysis of the relationship between prognostic factors and clinical outcome

Variable	OR	95% CI	*p*
Corticosteroids	0.068	0.005–1	**0.05**
Hepatic	22	1.333–362.84	0.137

Reference categories: Positive/Yes; bold values represent *p* ≤ 0.05

## Discussion

The observed prevalence in the present study was 23.8%, a value relatively similar to other studies undergone in Portugal with prevalence values of 20.4% [75], 22.1% [76] and 27.1% [11]. The study sample included a variety of cases, from apparently healthy cats that were diagnosed with only mild anemia or thrombocytopenia in routine pre-surgical analyses, to clinically ill cats that presented to the hospital in a state of severe anemia that were diagnosed during the clinical work-up.

Analysis of the relationship between risk factors and feline *Mycoplasma* spp. infection identified a statistically significant relationship with FIV-positive animals. This relationship was also identified by other studies, which have proposed that the retrovirus’ immunosuppressive effect enables infections by additional etiological agents or that this observed association is a result of similar transmission routes [42, 77, 78].

An association between feline *Mycoplasma* spp. and the senior age group has also been observed, with seniors appearing to be less likely to be IgM-positive cases. Feline mycoplasmosis tends to be more prevalent in adult animals due to the cumulative risk of exposure, but there are also studies that identified younger cats as more likely to be affected, as they tend to manifest more overt clinical signs [53, 55, 79]. This negative correlation with older cats may be due to a lower tendency for these animals to acquire recent infectious diseases as they’re less likely to leave home or engage in aggressive interactions that promote the transmission of haemoplasmas, compared to young cats who have a more active lifestyle, although different results could be conditioned by the way age ranges are defined for each study [44, 45, 80].

Despite being identified as relevant by other studies, collected data did not allow the identification of a relationship between infection and “typical” risk factors such as sex, reproductive status, lifestyle or origin of the cats [61, 71, 79, 81]. The absence of a correlation between feline haemoplasmas and the application of external parasiticides, or the presence of ectoparasites, suggests that transmission of these agents between cats in an urban area is not restricted to the activity of arthropod vectors [34, 44, 82].

The simultaneous detection of haemoplasma genetic material in vectors and feline hosts, from which they were removed, confirms their ability to ingest *Mycoplasma* spp. through their hematophagous activity [5, 10, 66, 83, 84], but does not imply that they are still viable to be transmitted to subsequent vertebrate hosts [14, 34, 85]. However, the frequently observed relationship between feline mycoplasmosis cases and cats that are also ectoparasite carriers, or that have not received adequate external parasiticides, can be a reason to consider the existence of vector-borne transmission [10, 17, 81, 86].

On the other hand, there are registered cases of feline mycoplasmosis in cats not infested with ectoparasites or cats that don’t even have access to the outdoors [87, 88], and although multiple attempts have been made, experimental transmission studies with vectors have provided inconclusive results [83, 89, 90]. Despite an apparent association of feline mycoplasmosis cases with animals infested with ectoparasites, this relationship may only represent a population of cats that have more access to the outdoors and are therefore more exposed to arthropod vectors or other cats of unknown health status [91]. Furthermore, there is recent evidence of canine haemoplasma transmission in an isolated group of dogs, all of which received proper ectoparasiticides, suggesting that haemoplasma transmission is not strictly dependent on the presence and activity of arthropod vectors [92].

Univariate analysis of the effect of acute feline mycoplasmosis infection on clinical, hematological, and biochemical variables found no statistically significant associations. However, multivariate analysis identified some positive trends between infected cases and the presence of anisocytosis or lymphadenopathy, nonspecific signs of disease that have also been identified by other authors [47, 48, 57]. Although not statistically significant, a negative correlation was observed between infected animals with low ALT or decreased total protein, which may be a mirror image of the increased liver enzymes and hyperproteinemia reported in acute cases of feline mycoplasmosis [56, 57].

Akin to other studies, no obvious association was detected between haemoplasma infection and complete blood count abnormalities associated with anemia [9, 66, 79]. This tenuous relationship with clinical and analytical signs can be explained by the complex dynamics between each cat’s individual response and inherent characteristics of the infecting species, which may translate into limited symptomatology that’s nonspecific and short-lived [21, 61, 93]. Another possible cause of these sparse results may be a higher proportion of cats infected by species with lower pathogenic potential such as “*Ca*. M. haemominutum” and “*Ca*. M. turicensis”, although it was not possible to distinguish between the three species due to the retrospective nature of the analysis [11–14].

Some animals show an effective response, with hematocrit rise and spontaneous elimination of the etiological agent, while other cats require therapeutic intervention to prevent progression to fatal hemolytic anemia, with metabolic acidosis and cardiovascular collapse [45, 66, 91, 94–96]. Antibiotics such as doxycycline or marbofloxacin have proven to be effective in reducing haemoplasma loads and help to hasten the recovery, but pharmacological therapy does not reliably allow for a complete elimination of the etiological agent [46, 69, 97].

The analysis of factors capable of affecting clinical outcome was only statistically significant with the use of corticosteroids, but a markedly positive trend was observed between the likelihood of recovery with the use of doxycycline. Meanwhile, clinical recovery registered a negative trend with the presence of concomitant diseases (of hepatobiliary or oncological nature). The negative relationship between a favorable prognosis and the use of corticosteroids is justified by the existence of more complex hemolytic anemia cases that didn’t respond to the administration of antibiotics, which led to glucocorticoids being added to the treatment plan even though their effectiveness in cases of mycoplasmosis is still debatable [61, 98].

The negative correlation of a positive clinical outcome with hepatobiliary or oncological disease is also an expected result as the presence of concomitant diseases is generally considered a negative prognostic factor [45, 61, 65]. On the other hand, the use of doxycycline has shown an obvious trend with favorable clinical resolution, proving its effectiveness and confirming its status as the first choice for an initial approach to feline mycoplasmosis [5, 47, 69].

This analysis of prognostic factors focused only on IgM-positive cases that were considered for treatment, but it should be noted that there was a portion of IgM-negative but IgG-positive patients who also received antibiotics after diagnosis. Seeing as these were cases with other prominent clinical conditions that may constitute a confounding effect on the studied prognostic factors, these “chronic” cases were not included as an attempt to perform an analysis focused on the outcome of acute mycoplasmosis.

This study was hampered by a few limitations, the main difficulty being the lack of information regarding the species of *Mycoplasma* affecting each patient and the inability to distinguish cases of co-infection from single infection cases. Another difficulty was the lack of standardization derived from the use of analyses performed by different laboratories, which, in addition to different equipment, use different reference values. Due to the study’s retrospective nature, as clinical cases were being collected, patient records already provided the case’s progress and outcome, but gaps in records resulted in missing data such as breed, vaccination status, internal and external parasite prophylaxis frequencies and applied drugs, origin of the cats, etc.

## Conclusions

The results of this study confirm the presence of haemotropic *Mycoplasma* spp. in feline patients treated at a veterinary hospital in Lisbon. Their presence in apparently healthy chronic carriers but also clearly anemic cats confirms the complex clinical and epidemiological dynamics of these agents. A positive serological or molecular result doesn’t always imply active disease, so it must be evaluated with the patient’s general condition, clinical signs, hematological and/or biochemical abnormalities, as well as concomitant or future factors that may trigger disease. The bacteria responsible for feline mycoplasmosis are present and circulate in the feline population through transmission routes that are still not clearly defined, and their clearance is not guaranteed by administration of appropriate antibiotic courses. As such, adequate monitoring of these agents should be maintained in animals that live in close contact with humans, given the lack of knowledge regarding their zoonotic potential.

## Data Availability

The datasets generated and analyzed during the current study are not publicly available to protect study participant privacy but are available from the corresponding author on reasonable request.

## Declarations

### Authors’ information

CIISA - Centro de Investigação Interdisciplinar em Sanidade Animal, Faculdade de Medicina Veterinária da Universidade de Lisboa; AL4AnimalS - Laboratório Associado para a Ciência Animal e Veterinária, Faculdade de Medicina Veterinária da Universidade de Lisboa, Portugal. Wenjun Li. CIISA - Centro de Investigação Interdisciplinar em Sanidade Animal, Faculdade de Medicina Veterinária da Universidade de Lisboa; AL4AnimalS - Laboratório Associado para a Ciência Animal e Veterinária, Faculdade de Medicina Veterinária da Universidade de Lisboa, Portugal. Ana Teresa Severino Caldeira Reisinho: CIISA - Centro de Investigação Interdisciplinar em Sanidade Animal, Faculdade de Medicina Veterinária da Universidade de Lisboa; AL4AnimalS - Laboratório Associado para a Ciência Animal e Veterinária, Faculdade de Medicina Veterinária da Universidade de Lisboa, Portugal. Luís Manuel Madeira de Carvalho.
